# Seasonally-Dynamic Presence-Only Species Distribution Models for a Cryptic Migratory Bat Impacted by Wind Energy Development

**DOI:** 10.1371/journal.pone.0132599

**Published:** 2015-07-24

**Authors:** Mark A. Hayes, Paul M. Cryan, Michael B. Wunder

**Affiliations:** 1 Department of Integrative Biology, University of Colorado Denver, Denver, CO, 80204, United States of America; 2 U.S. Geological Survey, Fort Collins Science Center, Fort Collins, CO, 80526, United States of America; University of Regina, CANADA

## Abstract

Understanding seasonal distribution and movement patterns of animals that migrate long distances is an essential part of monitoring and conserving their populations. Compared to migratory birds and other more conspicuous migrants, we know very little about the movement patterns of many migratory bats. Hoary bats (*Lasiurus cinereus*), a cryptic, wide-ranging, long-distance migrant, comprise a substantial proportion of the tens to hundreds of thousands of bat fatalities estimated to occur each year at wind turbines in North America. We created seasonally-dynamic species distribution models (SDMs) from 2,753 museum occurrence records collected over five decades in North America to better understand the seasonal geographic distributions of hoary bats. We used 5 SDM approaches: logistic regression, multivariate adaptive regression splines, boosted regression trees, random forest, and maximum entropy and consolidated outputs to generate ensemble maps. These maps represent the first formal hypotheses for sex- and season-specific hoary bat distributions. Our results suggest that North American hoary bats winter in regions with relatively long growing seasons where temperatures are moderated by proximity to oceans, and then move to the continental interior for the summer. SDMs suggested that hoary bats are most broadly distributed in autumn—the season when they are most susceptible to mortality from wind turbines; this season contains the greatest overlap between potentially suitable habitat and wind energy facilities. Comparing wind-turbine fatality data to model outputs could test many predictions, such as ‘risk from turbines is highest in habitats between hoary bat summering and wintering grounds’. Although future field studies are needed to validate the SDMs, this study generated well-justified and testable hypotheses of hoary bat migration patterns and seasonal distribution.

## Introduction

Effective conservation and management of migratory animals requires knowledge about the dynamics of habitat use across time and space, and about the migratory connectivity of populations [1]. Seasonal distribution and migration patterns are relatively well known for many bird species, especially those that are hunted for food, are of interest to bird enthusiasts, or that have large and conspicuous populations [2]. However, because most bats are small, reclusive, and rarely active or easily observable during the daytime, comparatively little is known about the seasonal distribution and migration patterns of bats [3,4].

Factors influencing the seasonal distributions and migration patterns of birds and other conspicuous migratory animals are sometimes broadly understood and such knowledge can guide conservation and management programs [e.g., 5,6]. However, physiological and behavioral constraints have differently influenced the evolution of migration in temperate-zone bats and birds; relatively few bird species are capable of extended hibernation (e.g., multi-week torpor bouts; [7]). Without the option of extended hibernation, birds that winter in temperate zones with long periods of freezing temperatures and associated food shortages have evolved strategies for weathering the cold and finding food while remaining active, whereas other birds migrate to warmer places where food is still available and where they can spend less of their available energy keeping warm [7,8]. In contrast, most species of temperate-zone bats are heterothermic and can use torpor for periods spanning days to months. During winter, many species of bats can access and communally exploit caves, mines, rock crevices, building interiors, and other deeply recessed shelters that offer the consistently cold, humid, dark, and undisturbed conditions that make extended hibernation possible [7,8]. A few temperate-zone species of bats avoid these secluded, underground shelters and, like many birds, roost in trees throughout the year (hereafter ‘tree bats’) [9,10]. Trees offer less protection from seasonal changes in ambient weather, light, and disturbance, so tree bats are confronted with substantially more variability in environmental conditions than other temperate-zone bats. These differences in roosting behavior likely influenced the tendency of certain tree bats to migrate longer distances (>1,000 km) between summer and winter habitats than most other bats [4,8]. Long-distance migration in tree bats may be driven by seasonal changes in surface environmental conditions and food availability similar to those influencing many migratory animals. However, migratory tree bats also tend to be heterothermic and have the additional option of using torpor to escape unfavorable conditions [11], leading to the likelihood of migration behaviors unique to tree bats that may require new methods to uncover and new paradigms to understand.

Compounding the difficulty of discovering seasonal distributions and migration patterns of highly mobile, cryptic, nocturnal species that use torpor, such patterns might seasonally differ between the sexes within a bat species. Some researchers have proposed that sex-biased migration may occur in bats due to long reproductive delays (e.g., autumn and winter mating followed by delayed fertilization until spring) and the different needs of male and female bats during spring and summer. For example, Fleming and Eby [4] hypothesized that the higher energetic needs of females during pregnancy and lactation has resulted in a resource cost-benefit tradeoff where reproductive females migrate to habitats with favorable temperatures for thermoregulation and greater prey abundance, while males concurrently seek habitats that allow daily or occasional torpor use, yet may have less prey available. Thus, for females the additional energetic expense of moving longer distances to areas with higher prey availability and roosting resources with optimal thermal conditions for raising young may outweigh the energetic costs of migration [4]. These and other authors presented evidence scattered over decades of female tree bats migrating earlier and farther than males [4,11,12].

Several hypotheses have been proposed regarding the continental migration patterns of bats [4,10,12–14], yet many remain untested because of the general difficulties of studying bat migration and the coarse-resolution of currently available methods for doing so [10,13]. We lacked a broader framework by which hypothesized seasonal distributions and migration patterns of migratory bats could be synthesized and evaluated. Unfortunately, this methodological shortcoming is becoming apparent in light of a pressing, practical need to better understand and characterize the seasonal distributions and migration patterns of migratory tree bats.

The effects of commercial-scale wind energy development on migratory wildlife populations are of increasing concern to ecologists and resource managers, because some species of song birds, raptors, and bats are consistently discovered dead beneath wind turbines in many parts of the world [15–17]. In terms of carcass numbers, bats are most abundantly found dead beneath wind turbines in North America [18,19]. Of the 45 species of bats that occur in the U.S. and Canada, four species of tree bats comprise the majority (>80%) of bat fatalities at commercial-scale wind energy facilities in those countries: hoary bats (*Lasiurus cinereus*), eastern red bats (*L*. *borealis*), western red bats (*L*. *blossevillii*), and silver-haired bats (*Lasionycteris noctivagans*) [20–23]. The reasons why these migratory tree bats form such a large proportion of fatalities at turbines in North America remain unknown [21].

Most bat fatalities at turbines in the U.S. and Canada occur during a period from about mid-July through October, which generally coincides with autumn migration [18,20]. Recent estimates suggest that wind energy facilities could be resulting in as many as hundreds of thousands of tree bat deaths in North America each year [17,19,22,24], far exceeding previously documented sources of accidental death in these species [22]. This unprecedented level of fatality at turbines comes at a time when population sizes of affected tree bats are unknown [25]. Uncertainty about how long tree bat populations can sustain accidental losses at turbines, as well as the desire to avoid or minimize such losses, is driving the need to better understand patterns of seasonal occurrence and migration in migratory tree bats. In particular, more precise hypotheses and maps of potential seasonal distribution and migration patterns are needed.

Hoary bats represent the largest proportion (approximately 40–50%) of documented bat fatalities at turbines in the U.S. and Canada [18,20,23]. Despite being one of the most wide-ranging mammals in the Americas and being considered a habitat generalist [26], hoary bats are infrequently observed and specific geographic distributions and habitat needs of the species during most seasons remain unknown. For example, the winter roosting behaviors and thermoregulatory strategies of hoary bats have not been discovered and well characterized, nor have their particular behaviors and habitat needs during migration periods [27]. Furthermore, like many other species of bats, adult male and female hoary bats exhibit distinct differences in seasonal thermoregulatory strategies and distributions [10–12]. Understanding such sex-based differences in distribution, thermoregulatory strategies, and how they change across seasons may shed light on the underlying drivers of habitat use by hoary bats, as well as help guide conservation and management efforts directed at their populations.

This study had two objectives. The first objective was to use species distribution modeling for the first time to try to better understand the sex-specific seasonal distributions of a highly mobile, flying mammal. With minimal precedent on which to base our approach, we used a suite of species distribution models to create maps of potential seasonal habitat use and, by consolidation, possible migratory patterns of hoary bats in North America. Species distribution modeling has been used to address many problems in theoretical and applied ecology [28,29], including evaluating ecological hypotheses, assessing the potential impacts of land use and climate changes, and suggesting sites with high potential for occurrence of rare or cryptic species [30–33]. Although species distribution models (SDMs) have the potential for revealing the structure of migratory connectivity for cryptic migratory species, little work has been done in this area (but see [34]). Because this is the first work of this kind, we used SDMs to articulate hypotheses rather than test them. We used five different SDM approaches, including logistic regression, multivariate adaptive regression splines, random forest, boosted regression trees, and maximum entropy, all of which have performed well in comparative analyses [28,29]. We developed a set of *a priori* predictor variables and combined the predictions from each modeling approach to generate ensemble models, which provide better predictive performance compared to a single modeling approach [28].

Prior to this effort, the only information available on the sex-specific seasonal distributions and migration movements of hoary bats at a continental scale were geographically coarse maps of occurrence locations [10,12], distant recaptures of marked bats [4], and estimated movements of individuals inferred from stable isotopes [13]. Various hypotheses had been proposed in the literature, yet no broader framework for articulating and summarizing such hypotheses existed. Therefore, our second objective was to interpret and present the ensemble maps generated by our SDMs as the first sex-specific, geospatially explicit hypotheses of seasonal habitat use, distribution, and migration patterns of hoary bats. We summarize by evaluating how the SDM model predictions relate to previously hypothesized continental migration patterns of hoary bats in North America, as well as how such model-formalized hypotheses might inform our understanding of important conservation issues like tree bat fatalities at wind turbines.

## Results

**Fig 1:**
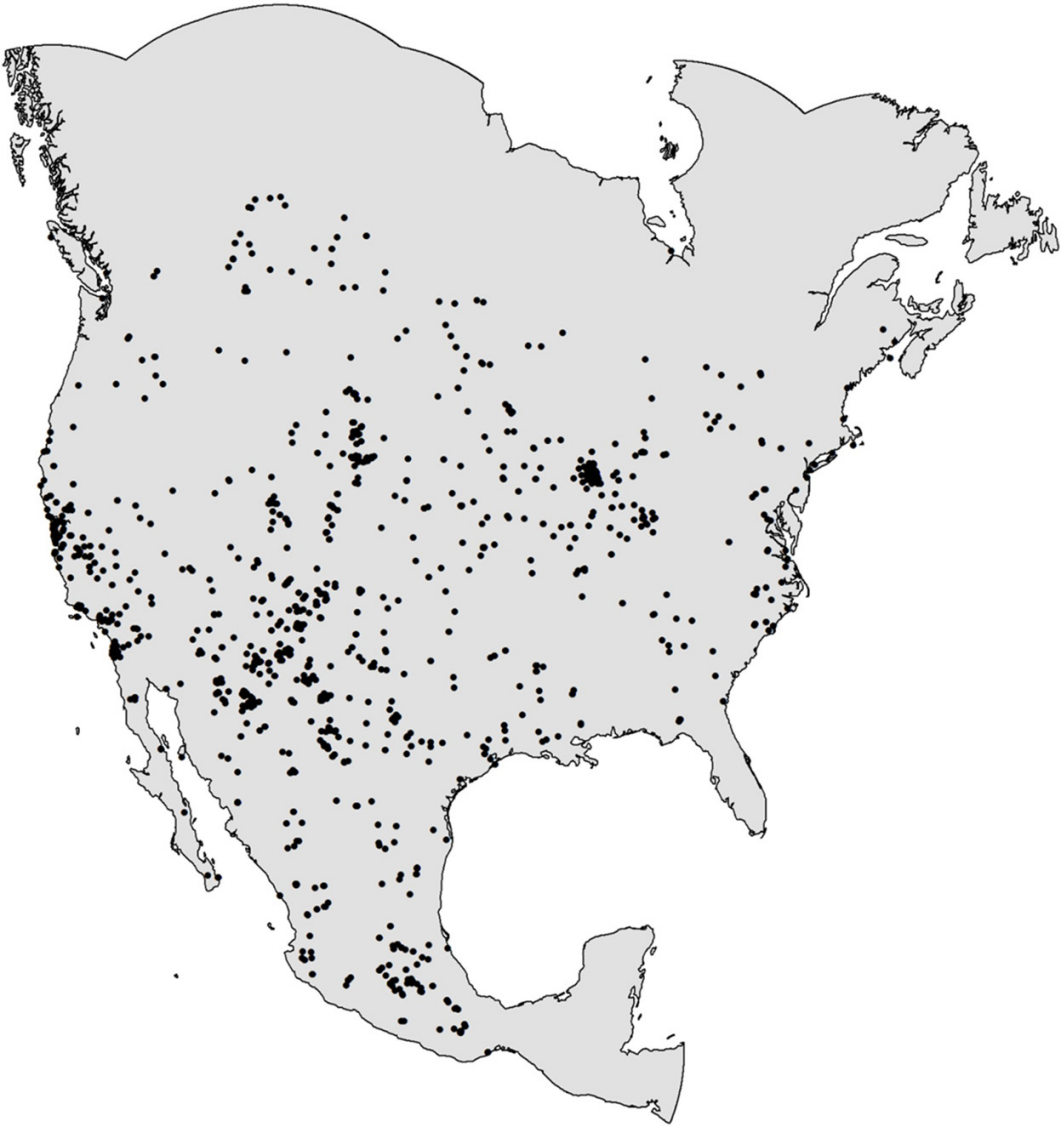
Spatial extent and occurrence record locations of hoary bats (*Lasiurus cinereus*) in North America, 1950–2000.

Of 3,215 hoary bat records documented between 1950–2000 in continental North America, we eliminated 462 records that identified locality to only state, province, or county, or that had an estimated spatial error after georeferencing of > 50 km (Fig 1). Species distribution models were fit to the remaining 2,753 occurrence records divided into 7 subsets, representing 1,068 occupied 1-km^2^ pixels: winter males and females (130 records, 61 locations), spring females (324 records, 165 locations), spring males (353 records, 89 locations), summer females (342 records, 182 locations), summer males (999 records, 239 locations), autumn females (240 records, 140 locations), and autumn males (365 records, 151 locations). SDM analyses were conducted with each subset of data using each of 5 model forms (generalized linear models (GLM), multivariate adaptive regression splines (MARS), boosted regression trees (BRT), random forest (RF), and maximum entropy (Maxent)), for a total of 35 model runs. The final sets of predictor variables used in this analysis are shown in Table 1.

**Table 1:** Final set of predictor variables used in modeling patterns of season distribution of hoary bats (*Lasiurus cinereus*) in North America.

Variable	Winter	Spring	Summer	Autumn
Mean seasonal temperature (°C)	X	X	X	X
Mean prior-season temperature (°C)	X	X	X	
Mean next-season temperature (°C)				X
Seasonal temperature range (°C)	X	X	X	X
Precipitation Seasonality (BIO 15)	X	X	X	X
MODIS Phenology EVI length of season (days)	X	X	X	X
MODIS Vegetation Continuous Field (VCF) tree cover (%)	X	X	X	X
Variables Used	6	6	6	6

### Species distribution model performance & ensemble mapping

**Fig 2:**
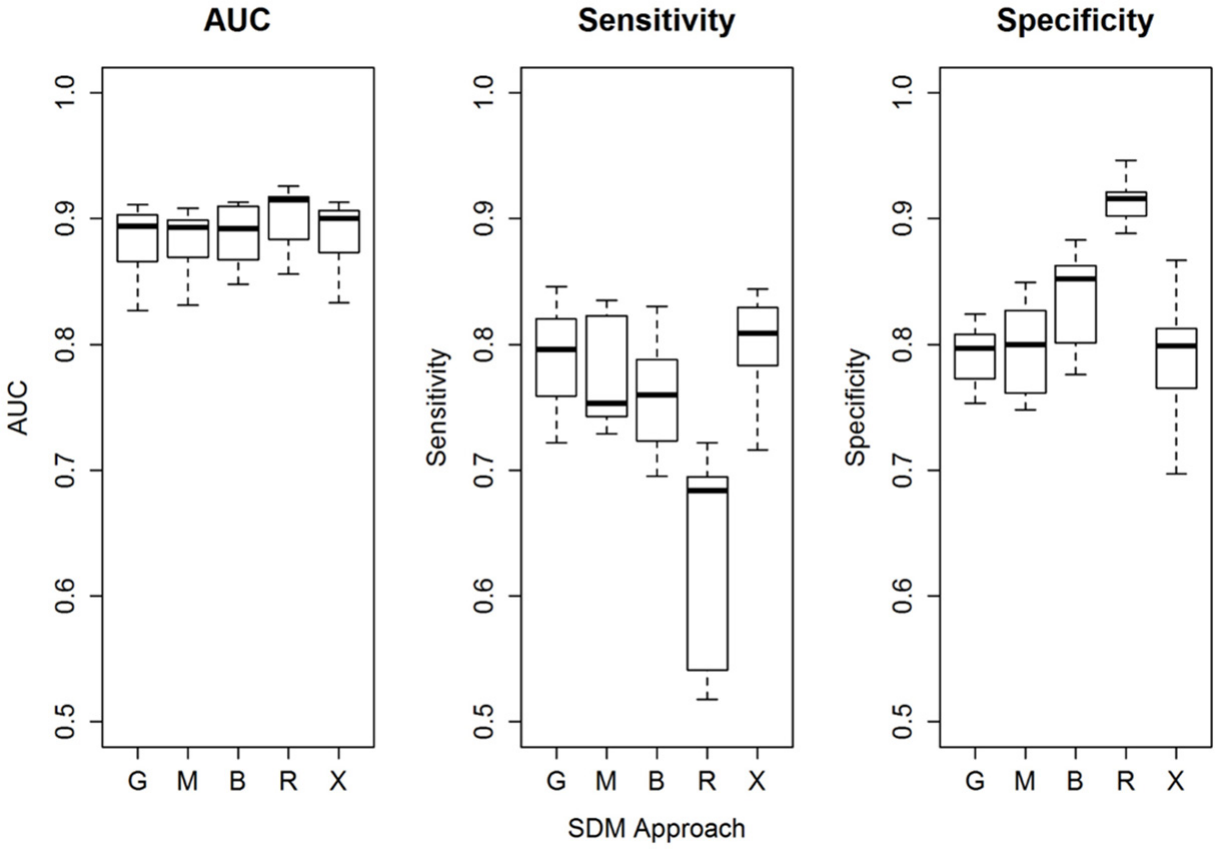
Boxplot comparing Area Under the Curve (AUC), sensitivity, and specificity statistics using test data and the cross-validation mean for each season and each of 5 forms using the final set of predictor variables in modeling patterns of season distribution of hoary bats (*Lasiurus cinereus*) in North America. G = Generalized Linear Models using logistic regression in a maximum likelihood framework; M = Multivariate Adaptive Regression Splines; B = Boosted Regression Trees; R = Random Forest; X = Maxent approach to maximum entropy modeling.

**Fig 3:**
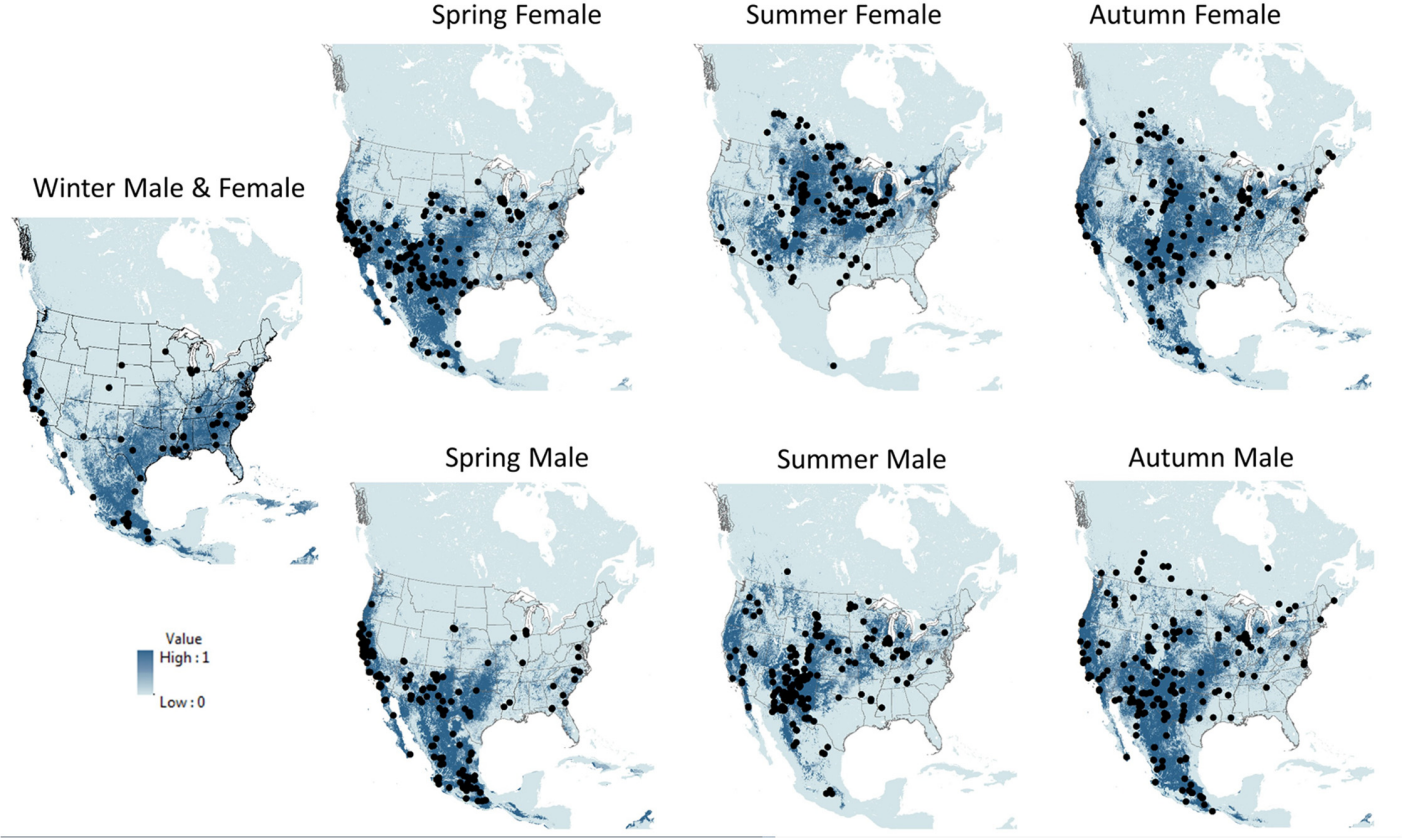
Ensemble maps for each season and sex for North American hoary bats (*Lasiurus cinereus*) using ensemble binary prediction from 5 species distribution model forms. Dots are occurrence record locations for hoary bats.

AUC scores ranged from 0.827–0.926, with little variation in AUC scores among model forms within a seasonal analysis (Table 2). RF was associated with the highest AUC score in every seasonal analysis; the lowest AUC scores were distributed among BRT, MARS, and GLM. Sensitivity (a model’s ability to predict true presences) ranged from 0.518–0.846 (Table 3). RF was associated with the lowest sensitivity in each seasonal analysis and the lowest mean sensitivity among all analyses; Maxent was associated with the highest sensitivity in 4 of 7 seasonal analyses, and the highest mean sensitivity among all models and all seasons (Table 3). With the exception of RF, sensitivities were similar among model forms within any single season analysis. Specificity (a model’s ability to predict back-ground cells) ranged from 0.745–0.946 (Table 3). RF was associated with the highest specificity in each seasonal analysis and the highest mean specificity among all models and analyses; Maxent was associated with the lowest specificity for 4 of 7 seasonal analyses, and the lowest mean specificity among all seasonal analyses (Table 3). With the exception of RF, specificities were similar among model forms within seasonal analyses. Variable importance for each season and model was ranked based on mean ΔAUC for each sex and season (Table 4). AUC, sensitivity, and specificity were similar across all models, except for RF which tended to have lower sensitivity and higher specificity compared to the other model forms (Fig 2). Considering the generally similar performance of all models and a lack of evidence that certain models chronically under-fit the location data, we chose to combine all model outputs in an ensemble approach rather than rely on any specific model. Ensemble maps for each of the 7 seasonal analyses are shown in Fig 3.

**Table 2:** Comparison of Area Under the Curve (AUC) statistic using test data and the cross-validation mean for each season and each of 5 species distribution model forms using the final set of predictor variables in modeling patterns of seasonal distribution of hoary bats (*Lasiurus cinereus*) in North America.

AUC for Each Season								
	Winter (♀♂)	Spring (♀)	Spring (♂)	Summer (♀)	Summer (♂)	Autumn (♀)	Autumn (♂)	Mean
GLM	0.911	0.895	0.911	0.881	0.894	0.851	0.827	0.881
MARS	0.902	0.893	0.908	0.892	0.895	0.846	0.831	0.881
BRT	0.892	0.885	0.913	0.907	0.912	0.848	0.850	0.887
RF	**0.917**	**0.908**	**0.926**	**0.917**	**0.915**	**0.856**	**0.859**	**0.900**
Maxent	0.913	0.900	0.913	0.893	0.900	0.853	0.833	0.886

Bold numbers indicate the highest AUC score for the season and sex. GLM = Generalized Linear Models using logistic regression in a maximum likelihood framework; MARS = Multivariate Adaptive Regression Splines; BRT = Boosted Regression Trees; RF = Random Forest; Maxent = Maxent approach to maximum entropy modeling.

**Table 3:** Comparison of sensitivity and specificity using test data and the cross-validation mean for each season and each of 5 species distribution model forms using the final set of predictor variables in modeling patterns of season distribution of hoary bats (*Lasiurus cinereus*) in North America.

Sensitivity/Specificity for Each Season								
	Winter (♀♂)	Spring (♀)	Spring (♂)	Summer (♀)	Summer (♂)	Autumn (♀)	Autumn (♂)	Mean
GLM	**0.846**/0.797	0.831/0.774	0.796/0.818	0.722/0.798	**0.809**/0.824	0.754/0.771	**0.764**/0.753	0.789/0.791
MARS	0.743/0.827	0.835/0.800	0.820/0.827	**0.825**/0.754	0.742/0.849	0.753/0.769	0.729/0.748	0.778/0.800
BRT	0.741/0.861	0.830/0.798	0.808/0.852	0.705/0.883	0.768/0.864	0.695/0.804	0.760/0.776	0.758/0.834
RF	0.526/**0.946**	0.684/**0.888**	0.722/**0.916**	0.699/**0.923**	0.690/**0.919**	0.518/**0.904**	0.556/**0.900**	0.628/**0.914**
Maxent	0.803/0.785	**0.836**/0.799	**0.844**/0.812	0.716/0.867	**0.809**/0.813	**0.823**/0.697	0.763/0.745	**0.799**/0.788

Bold numbers indicate the highest AUC score for the season and sex. GLM = Generalized Linear Models using logistic regression in a maximum likelihood framework; MARS = Multivariate Adaptive Regression Splines; BRT = Boosted Regression Trees; RF = Random Forest; Maxent = Maxent approach to maximum entropy modeling.

**Table 4:** Variable importance by season using increase in Area Under the Curve (AUC) when each predictor variable is permuted using 5 SDMs to model seasonal distributions of hoary bats (*Lasiurus cinereus*) in North America.

Season	Variable	GLM	MARS	BRT	RF	Maxent	Δ AUC
Winter (♀♂)	Season Length	0.25	0.22	0.19	0.15	0.11	0.18
	Temp Seasonal Range	0.25	0.05	0.06	0.07	0.12	0.11
	Tree Cover	0.06	0.04	0.07	0.04	0.05	0.05
	Autumn Temp	0.10	0.02	0.04	0.02	0.02	0.04
	Winter Temp	0.08	0.01	0.00	0.01	0.03	0.03
	Precip Seasonality	0.02	0.02	0.02	0.02	0.01	0.02
Spring (♀)	Season Length	0.19	0.22	0.18	0.14	0.15	0.18
	Spring Temp	0.18	0.03	0.15	0.10	0.07	0.11
	Tree Cover	0.08	0.05	0.00	0.08	0.07	0.06
	Winter Temp	0.02	0.03	0.00	0.03	0.01	0.02
	Temp Seasonal Range	0.00	0.02	0.00	0.03	0.03	0.02
	Precip Seasonality	0.01	0.01	0.00	0.04	0.02	0.02
Spring (♂)	Season Length	0.09	0.18	0.18	0.12	0.13	0.14
	Precip Seasonality	0.20	0.09	0.13	0.10	0.10	0.12
	Winter Temp	0.35	0.01	0.05	0.02	0.07	0.10
	Spring Temp	0.29	0.01	0.00	0.03	0.03	0.07
	Temp Seasonal Range	0.03	0.01	0.00	0.02	0.04	0.02
	Tree Cover	0.01	0.01	0.00	0.02	0.02	0.01
Summer (♀)	Summer Temp	0.35	0.28	0.07	0.07	0.31	0.22
	Spring Temp	0.28	0.17	0.05	0.08	0.28	0.17
	Season Length	0.12	0.14	0.09	0.09	0.14	0.12
	Temp Seasonal Range	0.12	0.04	0.10	0.13	0.05	0.09
	Precip Seasonality	0.04	0.03	0.00	0.03	0.05	0.03
	Tree Cover	0.00	0.00	0.08	0.03	0.02	0.03
Summer (♂)	Spring Temp	0.14	0.04	0.14	0.21	0.15	0.14
	Season Length	0.14	0.14	0.12	0.11	0.14	0.13
	Temp Seasonal Range	0.12	0.10	0.08	0.03	0.08	0.08
	Summer Temp	0.05	0.08	0.03	0.04	0.07	0.05
	Precip Seasonality	0.05	0.05	0.07	0.05	0.05	0.05
	Tree Cover	0.02	0.01	0.00	0.03	0.02	0.02
Autumn (♀)	Season Length	0.19	0.14	0.18	0.17	0.22	0.18
	Autumn Temp	0.15	0.08	0.15	0.07	0.16	0.12
	Winter Temp	0.18	0.07	0.00	0.03	0.21	0.10
	Tree Cover	0.06	0.04	0.09	0.08	0.07	0.07
	Precip Seasonality	0.08	0.04	0.05	0.06	0.07	0.06
	Temp Seasonal Range	0.12	0.03	0.00	0.03	0.08	0.05
Autumn (♂)	Season Length	0.17	0.13	0.14	0.17	0.17	0.16
	Precip Seasonality	0.13	0.03	0.07	0.08	0.09	0.08
	Autumn Temp	0.00	0.07	0.08	0.11	0.04	0.06
	Temp Seasonal Range	0.08	0.02	0.03	0.02	0.05	0.04
	Tree Cover	0.00	0.02	0.07	0.06	0.03	0.04

GLM = Generalized Linear Models using logistic regression in a maximum likelihood framework; MARS = Multivariate Adaptive Regression Splines; BRT = Boosted Regression Trees; RF = Random Forest; Maxent = Maxent approach to maximum entropy modeling.

#### Winter models

Of the variables used in the winter analysis (both sexes combined), growing season length had the highest mean increase in AUC (ΔAUC = 0.18), followed by winter temperature range (ΔAUC = 0.11). All SDM forms suggested that potential habitat suitability improved as growing season length increased beyond 200 days, with all model forms except Maxent predicting maximum habitat suitability with growing season length of 250–275 days. All model forms suggested that habitat suitability improved in areas with lower winter temperature ranges, where the difference between the maximum and minimum seasonal temperature is <20°C. The winter ensemble map suggests that male and female hoary bats might find favorable winter habitat along both coastlines and across large areas of the southcentral and southeastern United States, as well as much of Mexico (Fig 3).

#### Spring models

In the spring analysis of females, growing season length had the highest mean increase in AUC (ΔAUC = 0.18), followed by spring temperature (ΔAUC = 0.11). As with the winter analysis, all SDM forms suggested that habitat suitability improved as growing season length increased beyond 200 days, with all model forms except Maxent predicting maximum habitat suitability with a growing season length of 250–275 days. All model forms suggested that habitat suitability improved in areas with mean spring temperatures of 10–20°C. In the spring analysis of males, growing season length had the highest mean increase in AUC (ΔAUC = 0.14), followed by precipitation seasonality (ΔAUC = 0.12), and winter temperature (ΔAUC = 0.10). All SDM forms suggested that habitat suitability improved as growing season length increased beyond 200 days; habitat suitability also improved in areas with higher precipitation seasonality, with most models predicting that habitat suitability increased with increasing variation in seasonal precipitation. Spring ensemble maps suggest that female hoary bats might encounter broader regions of favorable habitat farther north in the continental interior than males during that season (Fig 3).

#### Summer models

In the summer analysis of females, summer temperature had the highest mean increase in AUC (ΔAUC = 0.22; this was the highest ΔAUC in all analyses), followed by spring temperature (ΔAUC = 0.17), and season length (ΔAUC = 0.12). All model forms suggested that habitat suitability improved in areas with mean summer temperatures > 15°C, and BRT and RF suggested that maximum habitat suitability occurred with mean summer temperatures between 15–25°C. All model forms suggested that habitat suitability for females during summer improved in areas with mean spring temperatures < 20°C, and BRT and RF suggested that maximum habitat suitability occurred with mean summer temperatures between 0–20°C. All SDM forms suggested that summer habitat suitability for females improved as growing season length increased beyond 150 days. In the summer analysis of males, spring temperature had the highest mean increase in AUC (ΔAUC = 0.14), followed by season length (ΔAUC = 0.13). All SDM forms suggested that maximum habitat suitability for males occurred in areas with spring temperatures between 0–20°C, and with a growing season length of > 150 days. Summer ensemble maps suggest that favorable habitat for female hoary bats might extend farther north and be more restricted to the interior of the continent than males; ensemble maps suggest proportionally less suitable habitat for males in the interior of the continent during summer, but more habitat than females in western areas of the U.S. and mountains of Mexico (Fig 3).

#### Autumn models

In the autumn analysis of females, growing season length had the highest mean increase in AUC (ΔAUC = 0.18), followed by autumn temperature (ΔAUC = 0.12), and winter temperature (ΔAUC = 0.10). All SDM forms suggested that habitat suitability improved for females in autumn as season length increased beyond 200 days, and in areas with mean autumn temperatures of 5–25°C. All model forms except Maxent suggested that maximum habitat suitability occurs in areas with mean autumn temperatures of about 15°C. In the autumn analysis of males, growing season length had the highest mean increase in AUC (ΔAUC = 0.16). All SDM forms suggested that habitat suitability improved as growing season length increased beyond 200 days, with all model forms predicting maximum habitat suitability in areas with a growing season length of 250–275 days. Autumn ensemble maps suggest a general similarity between sexes in the distribution of potential suitable habitat, with modeled areas of suitable habitat for both sexes covering greater expanses than during other seasons (Fig 3). The possible exception to the general similarity in potential suitable habitat between sexes in autumn is the comparative sparsity of potential suitable habitat for males in the northern Rocky Mountains and adjacent plains in Canada (Fig 3).

#### Wind turbine data

**Fig 4:**
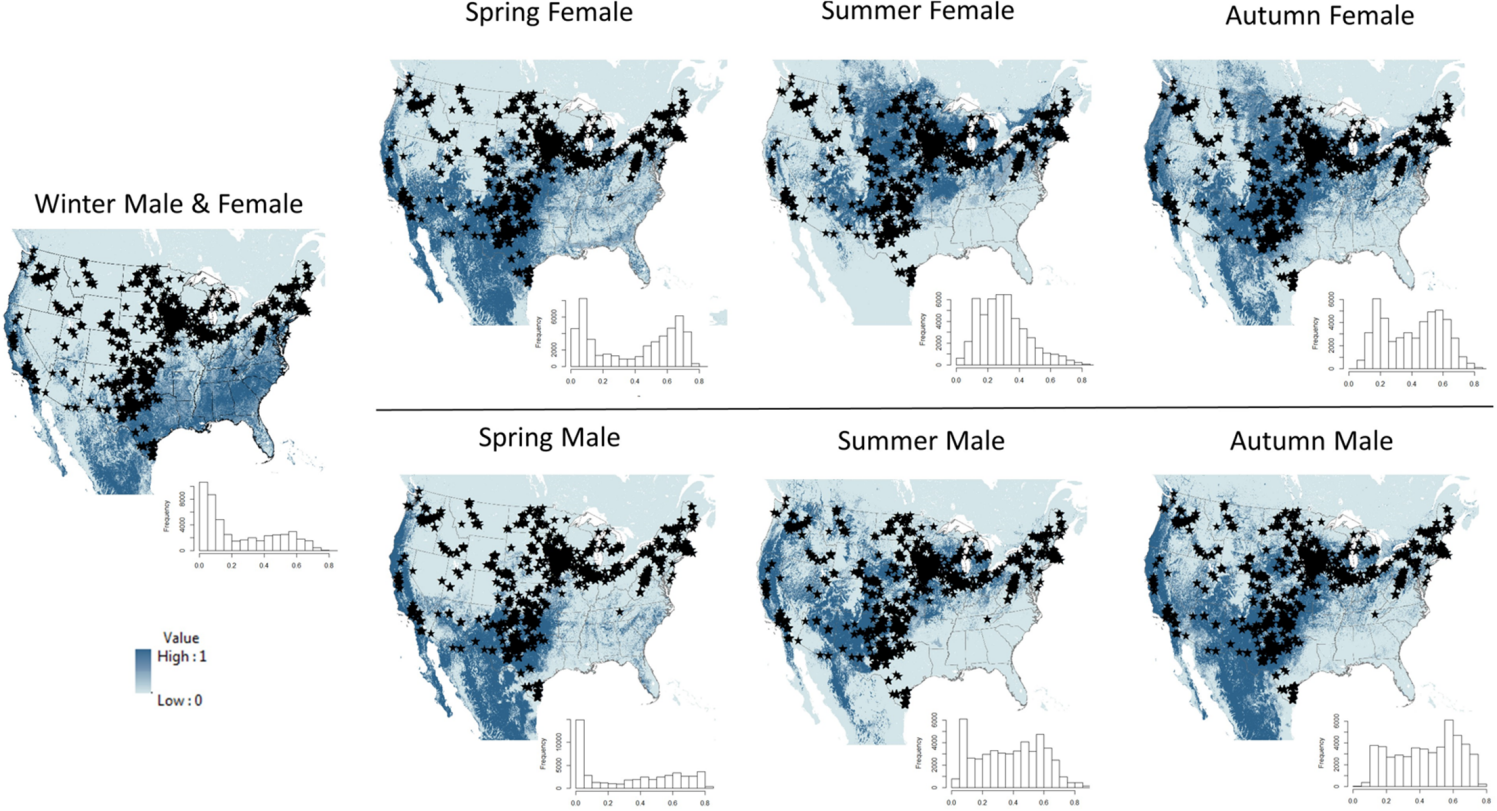
Ensemble maps for each season and sex for hoary bats (*Lasiurus cinereus*) in the United States using ensemble binary prediction from 5 species distribution model forms. Locations of > 47,000 industrial wind turbines in the contiguous United States are indicated by black stars. The color ramp indicates the proportion of the 5 SDM model runs for each season that are considered suitable habitat. Darkest blue indicates that all of the models predict the area to be suitable habitat, while lightest blue indicates that all of the models predict the area to be unsuitable habitat. The histogram associated with each map shows the empirical distribution for mean habitat suitability for all wind turbines in the United States, estimated using the 5 SDM approaches. Negative (left) skewness in the histograms, or higher frequencies of values toward the right side (= 1) of the horizontal axis, indicate overlap between modeled suitable habitat and turbine locations.

Maps of each seasonal ensemble map and the locations of 47,687 onshore industrial-scale wind turbines for the U.S. (and contained within our template) are shown in Fig 4. Mean seasonal habitat suitability for all wind turbines in our study area are: winter $\bar{x}$ = 0.247 (range 0.005–0.817); spring female $\bar{x}$ = 0.386 (range 0.003–0.805); spring male $\bar{x}$ = 0.340 (range 0.002–0.832); summer female $\bar{x}$ = 0.299 (range 0.013–0.851); summer male $\bar{x}$ = 0.379 (range 0.013–0.884); autumn female $\bar{x}$ = 0.407 (range 0.049–0.878); and autumn male $\bar{x}$ = 0.439 (range 0.035–0.772). Histograms showing the empirical distribution of mean ensemble habitat suitability for each season and sex at all wind turbine locations contained within the template are also shown in Fig 4. In general, the greatest overlap in potential suitable habitat for hoary bats and the location of existing wind turbines (negative [left] skewed histograms of Fig 4) occurs during autumn.

## Discussion

This was the first attempt to statistically model and map the potential sex-specific seasonal habitats of a wide-ranging migratory bat. Our results demonstrate how comparative species distribution modeling and ensemble mapping can be useful tools for generating well-justified hypotheses of animal distributions across seasons.

### Caveats and methodological considerations

While interpreting these modeling results it is helpful to know that the data on which our models were based come from museum specimens that can be biased in several ways, including geographic variability in: the efficiency of capturing individuals, torpor use and thus detectability, density of humans likely to sample and haphazardly encounter individuals, and differences in collecting practices [10,35–37]. These occurrence records represent presence only and not true absence. Our models also did not consider how the distributions of other species of bats might influence those of hoary bats. Because of the uncertainty introduced by these sources of bias and unaccounted-for factors potentially influencing habitat use, we emphasize that our model results and maps represent hypothetical distributions of hoary bats based on the places where they have been positively (not negatively) documented, not actual distributions. For example, if hoary bats actually occur in the boreal forests of Canada more often than our sample indicated, those habitats will be underrepresented in the model results and maps. Future efforts could improve upon our findings by including data gathered in less biased ways and by incorporating other plausible biotic and abiotic predictor variables into the models. Despite these assumptions and caveats, our models and maps represent the best sex- and season-specific distributional information yet compiled for this species, and are a step forward in understanding the seasonal distributions and seasonal movements of the cryptic species of bat being adversely affected by wind turbines, and for which high quality presence-absence data do not yet exist.

### Hypothesized seasonal distributions of hoary bats

#### Winter

Convention suggests that hoary bats migrate south during autumn to escape cold northern winters [12,36–41]. Subsequent observations led to the conclusion that at least some hoary bats might hibernate in northern areas [26,42–44]. Cryan [10] concluded that hoary bats occurred most often during winter in California, Mexico, and scattered locations in coastal areas of the eastern U.S. In our analysis, potential habitat suitability increased in areas with long growing seasons (>200 days) and temperature ranges of < 20°C. The winter ensemble map suggests great expanses of potentially suitable winter habitat throughout Mexico, along the Pacific Coast of the U.S., and across most of the south-central and eastern U.S. Our map of hypothesized winter distribution is consistent with earlier [10,12,38–41,45–48] and more recent [13] claims that warmer parts of North America provide suitable winter habitats for hoary bats. However, the general lack of occurrence records for hoary bats during winter from the south-central and eastern parts of the U.S. [10,12] raise the question of whether the broad expanses of potential habitat extending as far north as the Great Lakes that we modeled and mapped are actually occupied by hoary bats in winter. Given the conservation and management value of determining the true wintering grounds and other important seasonal habitats of hoary bats, areas where models predict habitat suitability but where evidence of species presence is lacking could be targeted for future presence-absence surveys to assess the validity of this seasonal hypothesis and further refine distributional understanding. The winter roosting behaviors and thermoregulatory strategies of hoary bats remain to be determined. Hoary bats can use torpor to save energy, at least for short periods of time, in air temperatures down to 0°C [11]. Although we do not know whether hoary bats remain active in winter, hibernate, or use an intermediate thermoregulatory strategy, our findings suggest they may select winter habitats that promote a combination of torpor and daily activity during warmer periods. Due to the relatively small number of winter records in our analysis, we pooled winter occurrence records of both sexes; however, we speculate that the habitat needs of male and female hoary bats may be similar during winter, and that males and females both require roosting conditions that are not excessively cold and that promote survival until spring. Testing for differences in the hypothesized winter habitat for males and females will require additional occurrence information from wintering bats.

#### Spring

Hoary bats have been captured in large numbers in western and southwestern parts of the U.S. during spring, presumably on migration [10,12,43,49,50]. Findley and Jones [12] presented evidence that female hoary bats began spring migration earlier than males and that females might move from wintering grounds in Mexico to eastern and northern parts of the continent during spring and early summer, whereas males seemed to migrate later and concentrate more in distribution in western areas of the continent during spring and early summer. Cryan [10] concluded that in addition to bats originating from wintering grounds in Mexico, some hoary bats observed in the southwestern U.S. during spring, and particularly females, were likely migrating eastward from California; analysis of occurrence records also provided little evidence that female hoary bats migrate northward out of California up the Pacific Coast during spring. Our maps of hypothesized spring distribution of males and females suggest that hoary bats tend to move through areas with relatively long (> 200 days) growing seasons and mean seasonal temperatures of 10–20°C during spring, but that they might not migrate through the northern Rocky Mountains and northern Great Plains in that season. It is unclear why spring temperature was an important variable in spring models for females only, or why potentially suitable habitat in the ensemble maps is more extensive for females than males during spring. Adult male hoary bats are more likely than females to use torpor in some areas during spring [11] and are assumed to migrate later than females, so differences in our models and maps could have been due to bias in detectability, sampling, or may reflect actual differential habitat selection between the sexes during spring. In general, our maps of hypothesized spring distributions of females and males indicate similar patterns, and suggest that hoary bats are transitioning during spring from wintering regions in maritime climates near the edges of the continent toward summering grounds near the interior of the continent, with greater expanses of contiguous potential spring habitat predicted for the western half of the continent. Our maps are consistent with prior hypotheses of spring movement by hoary bats out of wintering grounds in Mexico, California, and eastern coastal regions of the U.S. Despite evidence of movement toward the continental interior during spring, suitable habitat conditions may also persist in western wintering grounds (e.g., Mexico and California) into the spring. Our maps do not indicate that hoary bats are strongly associated with any mountain ranges during spring, although association with mountains seems to occur during summer and autumn.

#### Summer

Hoary bats have been observed throughout the U.S. and the southern half of Canada during summer [10,12,26], and several researchers have noted that they are not regularly documented in the southeastern U.S., east of the Mississippi River and south of the Ohio River [10,12,51]. Earlier studies presented evidence that adult males tend to be concentrated in distribution in mountainous areas of the western continent (including Mexico) during early summer, whereas females mostly occur east of the Rocky Mountains. Our maps of hypothesized distribution for both sexes predict potential summer habitat in areas with spring temperatures averaging < 20°C and with growing seasons of more than 150 days. Models for females included summer temperatures averaging > 15°C as having an important influence, but summer temperature was not an important predictor of male occurrence. Sex-specific differences in distribution, roost use, and thermoregulatory strategies are common in bats during summer [14,52], and it has been proposed that female hoary bats may be limited to areas of the continent with conditions favorable for gestation, lactation, and pup rearing, whereas males may not be as constrained by habitat needs during summer [11]. For example, our maps of hypothesized summer distribution indicate that California, with its seasonally dry Mediterranean climate, may not be suitable habitat for females during the summer months, but may be suitable for males. Our ensemble maps for summer females and males generally concur with prior understanding, although considerable areas of the western continent show potentially suitable habitat for female hoary bats, which contrasts the lack of previous evidence that female hoary bats regularly occur in western mountainous regions of the U.S. during summer. This is another area where our hypothesis maps differ from known patterns of distribution and could reflect incomplete characterization of the factors influencing the distribution of female hoary bats or the undetected presence of female hoary bats during summer across large swaths of the continent. Although these results suggest that females may occasionally travel farther between winter and summer grounds, they also suggest that there is considerable overlap in the summer distributions of male and female hoary bats.

#### Autumn

Earlier scientists concluded that hoary bats likely migrated south in the autumn to avoid colder northern winters and lack of available prey [12,38–40,53]. As more details on distribution emerged, others proposed that hoary bats may move toward coastal areas in autumn where winter temperatures are warmer than in the interior of the continent [10,12,13,45–48,54]. In our analysis, areas with growing season lengths of > 200 days were predicted to be suitable habitat for hoary bats during autumn, but average temperature was again only an important predictor in the models for females, suggesting females may be more likely to occur in areas with autumn temperatures that average between 5–25°C. The maps of hypothesized distribution during autumn suggest that contiguous expanses of potential habitat occur across much of the species’ range and over more of the continent than during any other season. Analysis of occurrence records previously indicated outward range expansion and coastward movements of hoary bats during autumn [10]. Although confirmatory studies are lacking, hoary bats are considered habitat and roosting generalists [26]. Our maps are consistent with the previously proposed hypothesis that hoary bats occur in more regions and habitat types during autumn than during any other season. With the possible exception of western Canada, where mostly female habitat is predicted, our maps also indicate fewer qualitative differences between the sexes in potential habitat during autumn compared to the other seasons for which sex-specific models were created. Hoary bats begin mating during late summer and autumn [55] and it is suspected that sex differences in distribution and habitat use are less prevalent in autumn as the species returns to wintering grounds. Similar to the presumably transitional habitats inferred from spring models, autumn maps of hypothesized distribution do not indicate that hoary bats would be unlikely to encounter abundant suitable habitat in southeastern coastal regions while moving from their summering areas toward wintering grounds during autumn.

#### Impacts of wind energy development

Migratory tree bats represent a substantial proportion (> 75%) of bat fatalities at wind energy facilities, and hoary bats represent the largest fraction (approximately 40–50%) of all documented bat fatalities in North America [15,17,20,23]. Our results offer new insight and testable hypotheses about where important seasonal habitats of hoary bats may occur and the areas through which they may travel among seasons. Our model results and maps are not suitable for predicting the actual presence of hoary bats, but represent hypotheses of where the species may occur at any given time of year. We emphatically state that confirmatory field studies are necessary to assess the models and hypothesized distributions described herein, preferably by following the detailed movements of many individuals with tracking devices (e.g., satellite tags) or in a probabilistic sampling framework using presence-absence sampling methods. Most bat fatalities at wind energy facilities in the temperate zones of North America occur during late-summer and autumn [18,20] and our modeling results may help better determine where hoary bats are likely to occur during that period of susceptibility. Unfortunately, fatalities at turbines occur most during the season in which our models suggest that potentially suitable habitats for hoary bats span across broader areas of the continent than during any other time of year. If these hypothesized distributions are confirmed, then hoary bats may be more seasonally general in their habitat use (or probability of being found out of their usual habitat) in autumn than during any other time of year, making it more difficult to predict the specific places they are likely to occur during the period of greatest susceptibility. However, alternative approaches to determining autumnal habitats of hoary bats may be possible.

Developing a better understanding of where hoary bats winter may be a key element of trying to predict and avoid fatalities at wind turbines. Our maps of hypothesized distribution indicate that hoary bats winter in locations with relatively long growing seasons and where winter temperatures are moderated by proximity to oceans. California and Mexico might be used consistently during winter, but the extent of wintering habitat in coastal regions of the eastern and southeastern U.S. remains to be determined due to disagreement between our winter model results and the lack of prior occurrence records for more interior regions of the southeastern U.S. during winter [10]. Future studies are needed to assess these new hypotheses of seasonal distribution, which may indicate that risk of wind energy to bats may be highest in habitats between interior summering areas and coastal wintering grounds (e.g., near passes in coastal mountain ranges). We predict that wind turbines built between the summering and wintering grounds of hoary bats will cause more fatalities during the period of susceptibility than those built out of transitional areas. Ironically, strategically sampling wind turbines at a continental scale for hoary bat fatalities and then comparing fatality numbers and sex composition to SDM-predicted, sex-specific habitat suitability may be a feasible method of evaluating our seasonal distribution and migration hypotheses. Only by such validation might the use of SDMs to predict fatality and avoid high-risk sites for wind energy development move from the realm of theoretical to practical.

## Materials and Methods

### Data for Modeling and Evaluation

We used museum occurrence records of hoary bats collected from 1950–2000 in North America originally compiled for another study [10]. This period was before the broad-scale deployment and reporting of bat fatalities at industrial wind turbines. The extent of the analysis included North America from the southern Mexico border with Guatemala to the northern coast of Alaska and Canada, with a 1,000-km buffer placed around the northernmost hoary bat occurrence locations (Fig 1). The extent was expressed as a raster with pixel resolution of 1 km^2^ in the North American Albers Equal Area Conic projection (NAD83 datum). We estimated the collection location and associated spatial error for each museum record in decimal degrees (latitude and longitude, reported to 3 decimal places; i.e., lat. = 42.348, lon. = -71.100). We eliminated occurrence records that had information identifying locality to only state, province, or county, or had an estimated spatial error after georeferencing of > 50 km. The occurrence data were segregated by reported calendar date of collection into seasonal categories as follows: winter, December 1 to February 29; spring, March 1 to May 31; summer, June 1 to July 31; autumn, August 1 to November 30. We used August 1 as the beginning date for autumn because we suspected that in some parts of the continent hoary bats began their autumn migration in early August. Background samples were generated by selecting a random sample of 10,000 pixels from all available pixels in the extent [56]. If a background sample coincided with an occurrence record, the pixel was classified as being occupied.

The predictor variables considered were selected based on their relevance as plausible predictors of hoary bat occurrence, but we restricted use of predictor variables to those that had a North American or global extent and an estimated resolution of approximately 1 km^2^. As potential predictor variables, we considered use of climate data (WorldClim minimum, maximum, and mean temperatures (°C) and precipitation (mm) from 1950–2000; www.worldclim.org; [57]), USGS topographic data (elevation (m); ned.usgs.gov [58]; MODIS Vegetation Continuous Field data (% bare ground, % tree cover, % herbaceous cover; collected 2000–20010; [59]; www.landcover.org/data/vcf/), and MODIS Phenology data (i.e. beginning of season (Julian date), length of ‘growing’ season (days), and greenup rate (vegetation index unit per 8 days); collected 2001–2007, except 2005; [60]; modis.gsfc.nasa.gov). Prior to final selection of predictor variables, we evaluated the correlation between each possible pair of predictor variables and eliminated one variable from each pair that was strongly correlated (Pearson or Spearman correlation, r > 0.70; [56]). We expected mean seasonal temperatures to influence hoary bat occurrence during all seasons, but that seasonal temperatures may be especially important during the winter period when hoary bats may avoid areas with prolonged very cold temperatures (e.g., < 0°C) that would make hibernation excessively challenging, especially given that hoary bats are thought to roost in the foliage of trees or above ground [26]. We also suspected that hoary bats might seek winter areas with moderate temperatures where use of hibernation throughout the winter may not be necessary. Conversely, because very hot temperatures (e.g., > 40°C) may threaten energy and water balance and reduce reproductive success, we suspected that female hoary bats might avoid areas with prolonged very hot temperatures during the spring and summer months when fetal development, parturition, lactation, and growth of newborns to volancy take place [11]. Thus, mean seasonal temperature was used for all seasonal models, where mean seasonal temperature for each pixel was calculated using monthly mean temperatures derived from monthly Worldclim temperature data (°C*10) from 1950–2000, and averaged for each season using the Raster Calculator function in ArcMap (Version 10.1, Esri software, Redlands, CA, USA). We also suspected that the prior-season temperatures for a given pixel might also influence the likelihood of the pixel being predicted as occupied in the current season. For example, temperature conditions during the autumn might influence the distribution and activity patterns of hoary bats in the winter (i.e. the decision to migrate or stay and hibernate at a given location might be influenced by autumn temperatures). Thus, we included prior-season mean temperature as a predictor variable in all seasonal models, and calculated the value for each pixel using monthly Worldclim temperature data (BIO1; °C*10) in the same way described above for the mean seasonal temperature. Elevation was highly correlated with mean annual temperature and seasonal temperature averages, and because surface temperatures do not follow a uniform cline with elevation [61], we assumed that surface temperatures have a stronger influence than elevation on continental distribution patterns of hoary bats. Thus, elevation was excluded from use in final model sets, while seasonal temperature averages were retained. Because of their tendency to roost in trees, hoary bats and other migratory tree bats are exposed to much wider variations in ambient and seasonal temperatures than are some species that roost in caves, mines, and rock crevices. Bats that roost inside caves, abandoned mines, and rock crevices often experience relatively stable temperatures across seasons and very little daily temperature variation [62,63], whereas tree-roosting bats experience substantial fluctuations in daily and seasonal ambient temperatures [64,65]. Thus, we considered using seasonal Worldclim diurnal temperature range data (derived from monthly BIO2 values; °C*10) and Worlclim temperature range data (derived from monthly BIO 7 values; °C*10). However, mean diurnal range values were highly correlated with MODIS Phenology EVI length of season (days) data (see below), while seasonal temperature range data were not. Thus, we included seasonal temperature range data (derived from monthly BIO7 values; °C*10) as a predictor variable in all seasonal models, and calculated the value for each pixel in the same way described above. We expected season length, the time from the start of the growing season to the end of the growing season (days), to positively influence hoary bat occurrence during all seasons. This is because we assumed that season length should influence insect prey availability and may result in improved fitness and survival due to the metabolic advantage of a lengthened period of prey and metabolic energy availability [66–69]. Thus, MODIS Phenology EVI length of season (days) data was included as a predictor in all seasonal models. Other MODIS Phenology data were not used due to high correlations with one or more variables already chosen. Annual precipitation (BIO 12; mm) and seasonal precipitation were eliminated as possible predictors due to high correlations with season length or other predictors. However, Worldclim precipitation seasonality (BIO 15; mm) was used as a predictor in all models. We also expected the availability of trees as roosting resources to influence hoary bat occurrence during all seasons. Thus, MODIS Vegetation Continuous Field (VCF) tree cover (%) was used in all seasonal models. We were interested in including relative humidity data in this analysis, but are not aware of such data at the spatial resolution and extent used in this study (1 km^2^ pixels in North America). The final sets of predictor variables used in this analysis are shown in Table 1.

### Modeling Methods and Evaluation

We analyzed seasonal distribution of male and female hoary bats using 5 different forms of species distribution models: generalized linear models using logistic regression and maximum likelihood estimation (GLM), multivariate adaptive regression splines (MARS), boosted regression trees (BRT), a random forest algorithm (RF), and maximum entropy (Maxent), using 10-fold cross-validation for each form [28,29,56]. These SDM forms are considered reasonably robust for other wide-ranging species [28,29,56,70–74].

Each model produced an estimate of potential habitat suitability for each pixel, expressed as continuous values between 0 and 1 and interpreted as the potential habitat suitability for a given pixel in North America. Using minimum training presence as a threshold for each model, we mapped potential habitat suitability for each season and model form using binary maps. Maps of potential hoary bat distribution for each season where generated using each model. Because of the small number of winter occurrence locations, males and females were pooled for the winter analysis. For display purposes, we generated 7 ensemble maps as follows: winter males and females, spring females, spring males, summer females, summer males, autumn females, and autumn males. The ensemble maps use the average of the binary estimates (0 or 1) of the 5 models for each pixel in the map. We used the Software for Assisted Habitat Modeling (SAHM) package for VisTrails software to fit species distribution models using GLM, MARS, BRT, RF, and Maxent, and also to calculate related performance metrics [75–77].

There are several performance metrics commonly used to compare SDM performance. To better understand comparative SDM performance among the 5 model forms, we use area under the receiver operating characteristic curve (AUC), sensitivity (a model’s ability to predict true presences), and specificity (a model’s ability to predict background cells)[78–80]. The AUC statistic is considered a threshold-independent approach to model evaluation [31,56,70,81–83]. For threshold-dependent metrics (sensitivity and specificity) we used the minimum training presence threshold [29]. We evaluated variable importance by season and by model using change in AUC statistics with and without the variable, but with all other variables in the final model used. We calculated increase in AUC (ΔAUC) when each predictor variable was permuted for each model and season, and then ranked predictor variables by mean ΔAUC [83]. Variable importance by season and model was calculated using increase in AUC (ΔAUC) after fitting the final model with and without each predictor variable in the final model [83]. Predictors for each season and sex were then ranked based on mean ΔAUC for each sex and season. If ΔAUC is relatively large this suggests that the predictor contributes substantially to the model, and likewise if the ΔAUC is relatively small this suggests that the predictor contributes less to the model.

Finally, on top of each seasonal ensemble map, we mapped the locations of approximately 47,000 onshore industrial-scale wind turbines for the United States (eerscmap.usgs.gov/windfarm/; accessed 5 October, 2014; [84]). We also extracted and created histograms of the ensemble habitat suitability for each season and sex at all wind turbine locations contained within the template. We used these maps and histograms to examine relationships between potentially suitable habitat for each sex and season and wind turbine locations for the United States. The occurrence data used in this analysis and an example VisTrails-SAHM file are available through the USGS’s ScienceBase Catalog (https://www.sciencebase.gov/catalog/item/55887bfde4b0b6d21dd61909; data deposited under doi:10.5066/F7WD3XM6).
